# Management of inflammatory bowel disease and serum level of infliximab in newborn exposed to anti-TNF therapy during pregnancy: Case report and literature review

**DOI:** 10.1097/MD.0000000000028274

**Published:** 2021-12-23

**Authors:** Maria Cecília de Aragão, Rodrigo Fedatto Beraldo, Mariana Barros Marcondes, Jaqueline Ribeiro de Barros, Giédre Soares Prates Herrerias, Rogerio Saad-Hossne, Júlio Pinheiro Baima, Ligia Yukie Sassaki

**Affiliations:** a Department of Internal Medicine, São Paulo State University (Unesp), Medical School, Botucatu, São Paulo, Brazil; b Department of Surgery, São Paulo State University (Unesp), Medical School, Botucatu, São Paulo, Brazil.

**Keywords:** biological therapy, case report, inflammatory bowel disease, pregnancy, ulcerative colitis

## Abstract

**Rationale:**

Heightened inflammatory bowel disease (IBD) activity during pregnancy is associated with higher rates of preterm birth, miscarriage, and low birth weight. Therefore, its adequate treatment is essential, considering the risk–benefit of medication use. Although previous literature has described the management of IBD during pregnancy, few studies have assessed the pharmacokinetics of IBD drugs in the newborn. In this case report, we describe the management of ulcerative colitis during pregnancy and discuss the benefits of checking serum levels of infliximab in newborns exposed to the medication during pregnancy.

**Patient concern:**

A 37-year-old patient with ulcerative colitis in clinical and endoscopic remission had been undergoing treated with infliximab since 2008. The patient became pregnant in 2018.

**Diagnosis and intervention:**

Infliximab medication was discontinued at the 29th week of pregnancy.

**Outcomes:**

The pregnancy was uneventful, and the levels of infliximab in the umbilical cord were >20 μg/dL. Live vaccinations were postponed until the baby was 6 months old, when a new serum drug level proved to be undetectable.

**Lessons:**

Our case suggests that the use of infliximab is safe in pregnancy, and drug discontinuation could be considered from the 24th week of pregnancy onward to reduce placental transfer to the newborn in patients at low risk of relapse. Vaccines with live attenuated organisms should be delayed for at least 6 months or until the serum level of the medication is undetectable.

## 1 Introduction

Inflammatory bowel diseases (IBDs) are highly prevalent in young people aged between 15 and 35 years. Thus, reproductive issues often arise during the course of the disease.^[1,2]^ Active disease at the time of conception is associated with higher rates of preterm birth, miscarriage, and low birth weight.^[2,3]^ It is recommended that pregnancy is attempted during remission and medications should be maintained, always considering the risk–benefit for the mother and baby.^[3]^

The use of infliximab, an anti-tumor necrosis factor (TNF) agent, is considered safe during pregnancy, and it could be discontinued at the 24th week of pregnancy to reduce placental transfer and associated neonatal immunosuppression,^[2]^ in patients at low risk of relapse. However, its discontinuation is associated with higher rates of disease relapse (36–39%) than with medication maintenance throughout pregnancy (25–26%).^[4]^

A systematic review that evaluated more than 1500 pregnant women exposed to anti-TNF therapy showed no increased risk of unfavorable pregnancy outcomes such as miscarriage, premature birth, low birth weight, and congenital malformations.^[4]^ However, newborns exposed to intrauterine anti-TNF agents, except certolizumab, should not receive live vaccines for 6 to 12 months or until the drug serum levels become undetectable to reduce the risk of active viral replication or vaccine failure.^[1,4–7]^ Live vaccines include tuberculosis (Bacille Calmette-Guérin - BCG), oral polio vaccine, measles, rotavirus, yellow fever, varicella-zoster, and influenza and are important strategies to protect against infections, especially in developing countries.

As the use of biological therapies for the treatment of IBD increases, some uncertainties remain regarding the management of pregnant patients on biological therapies, as well as the repercussions on maternal and fetal health. These uncertainties are accentuated by the fact that although the management of IBD during pregnancy has been discussed in the literature, the same is not true for the role of drug level monitoring in the newborn. Herein, we discuss the evolution and management of IBD in pregnant patients with ulcerative colitis (UC) and the benefits of monitoring serum levels of infliximab in newborns exposed to this medication during pregnancy, such as safety in the application of vaccines with live microorganisms in newborns.

## 2 Case report

The patient was a 37-year-old female diagnosis with UC and pancolitis. The patient was admitted to the emergency room in May 2008 for bloody diarrhea comprising eight bowel movements/day, abdominal pain, and fever. On physical examination, the patient appeared acutely ill and was found to be tachycardic, hypotensive, and febrile, with a distended and painful abdomen. Laboratory tests were significant for the following: hemoglobin, 9.2 g/dL (11.5–14.9); hematocrit, 28.6% (35.3–46.1%); and C-reactive protein, 31 mg/dL (<1). Abdominal radiography showed colonic dilatation of 6 cm. The patient was admitted to the intensive care unit owing to suspected toxic megacolon. She was treated with antibiotic therapy (ceftriaxone and metronidazole) and vigorous hydration. Sigmoidoscopy revealed severe UC flare. Histological examination confirmed UC and excluded *Clostridium* or cytomegalovirus infection. Treatment with 300 mg/day of intravenous hydrocortisone was initiated that had a good response. She was discharged from the hospital after 31 days of hospitalization on 40 mg/day of prednisone and 2 mg/kg/day of azathioprine. Due to the severity of the disease and poor response to azathioprine, combined therapy with infliximab was initiated with the first dose received in July 2008. The patient showed clinical and biochemical improvement with successful corticosteroid tapering. In February 2009, ileocolonoscopy showed normal terminal ileum, presence of pseudopolyps, and scarring mucosa in all colonic segments, compatible with endoscopic remission (endoscopic Mayo score: 0). The disease remained under adequate control on combination therapy until 2017, when the patient became pregnant, and azathioprine was suspended. She remained asymptomatic during pregnancy, and the last infliximab infusion was administered at 29th week of pregnancy. In August 2018, and while in the 38th week of gestation, she went into spontaneous labor and delivered vaginally a newborn weighing 2370 g (APGAR 9/9/9) without complications. At that time, umbilical cord blood sample was collected to measure the serum level of infliximab in the newborn, which was >20 μg/dL (3–5). The patient resumed infliximab infusions 2 months after delivery and has remained on monotherapy with complete disease remission. Repeat measurement of serum level of infliximab in the infant showed undetectable infliximab 6 months of age, and live attenuated vaccines were initiated.

The study was approved by the local Research Ethics Committee (CAAE: 43178720.6.0000.5411). Written informed consent was obtained from the patient for publication of this case report and any accompanying images.

## 3 Discussion

Although patients with IBD in remission are as fertile as the general population, fewer children are born to this population due to patient choice.^[1]^ Active disease impairs fertility, probably through multifactorial mechanisms such as pelvic inflammation, malnutrition, decreased libido, dyspareunia, and depression.^[8,9]^ Active disease at the time of conception, during pregnancy, and childbirth is associated with adverse events, such as premature birth, miscarriage, and low birth weight. Thus, conception is advised during disease remission.^[3]^

Regarding preconception care, the prescription of 400 to 800 μg/day folic acid in combination with adequate dietary intake is recommended to reduce the incidence of neural tube defects. This therapy must be started 1 month before conception and maintained during the first trimester. In women who use sulfasalazine chronically, doses higher than 2 mg/day are recommended.^[7]^

Regarding the effect of IBD-related medications during pregnancy, the use of thiopurines was not associated with complications of physical and mental development or the risk of infections during childhood. However, the association with the development of congenital anomalies is still debated,^[1]^ due wot which the medication was suspended in the reported patient. Regarding aminosalicylates, a small increase in the incidence of congenital malformations was observed in a meta-analysis published by Cornish et al,^[10]^ but there were confounding factors such as disease activity. Corticosteroids cross the placental barrier but are converted into less active metabolites, resulting in decreased levels in the fetal bloodstream.^[1]^ Some studies have shown that orofacial malformations in the newborns may complicate corticosteroid use during the first trimester of pregnancy^[11]^; however, another cohort with more than 50,000 pregnant women contradicts this finding,^[1]^ and more studies are needed to assess the real risk. The Pregnancy in Inflammatory Bowel Disease and Neonatal Outcomes study linked the use of corticosteroids to the incidence of gestational diabetes, premature birth, and low birth weight.^[12]^

Data on cyclosporine and tacrolimus are sparse. Prematurity and low birth weight have been reported but differentiating between disease severity and medications as the cause of these adverse events is difficult.^[1]^ Methotrexate and thalidomide are contraindicated in pregnancy because of their teratogenic effects.^[1]^

Regarding the use of vedolizumab, complications such as premature rupture of membranes, pre-eclampsia, abortion, and stillbirths have been reported in up to 25% of pregnancies and neonatal complications, including prematurity, intrauterine growth retardation, and congenital malformations in up to 35% of infants from mothers exposed to the medication.^[2]^ A more recent study^[13]^ confirmed an increase in unfavorable outcomes, such as premature births and spontaneous abortions in pregnant women taking vedolizumab, but the results may be related to disease activity. The same results were seen in a recently published meta-analysis,^[14]^ in which the prevalence of early pregnancy loss and preterm birth were higher in vedolizumab vs anti-TNF users. On the contrary, the study CONCEIVE^[15]^ showed no increased risk of maternal or fetal adverse results when comparing pregnancy and child outcomes between vedolizumab users to anti-TNF exposed or both immunomodulatory and biologic unexposed pregnancies.

With respect to ustekinumab, a meta-analysis published in 2021^[16]^ assessed outcomes in 43 pregnancies; however, 57% discontinued the medication before term and only 16 patients continued ustekinumab throughout pregnancy. There were no differences in the rates of congenital anomalies (2%), miscarriages (12%), or healthy live births (74%) compared with that in the general population.^[16]^ Placental transfer of tofacitinib has not been evaluated, but the medication has shown teratogenicity in animal models and should be discontinued 6 weeks before conception.^[2,17]^

In the study by Mahadevan et al,^[18]^ the 1-year infant outcomes of 1010 pregnancies were evaluated in which the mothers exposed to thiopurines (n = 242), biological agents (n = 642), or both (n = 227) were compared with those unexposed to drugs (n = 379). Drug exposure did not increase the rate of birth defects, miscarriages, premature birth, low birth weight, or infections during the first year of life. Thus, the authors concluded that medical therapy should be continued during pregnancy to maintain disease control.^[18]^ Other studies, such as the EVASION,^[19]^ did not show an increase in severe short-term or long-term infection in children exposed to intrauterine anti-TNF medications. However, the combination of anti-TNF and thiopurine therapy increased the risk of benign infections in infants compared with anti-TNF monotherapy alone.^[4,19–21]^ Because infliximab can be detected in infants up to 12 months after delivery,^[4]^ there is some concern about the effect on the immune system, rate of infection, and response to vaccines, especially with living organisms.

The largest prospective study in children exposed to immunomodulators or biologics^[22]^ showed that there was no association between exposure to medications and lower response rates to tetanus and *Haemophilus influenzae* B vaccines compared with in children of mothers with IBD unexposed to medications. Regarding the BCG vaccine, it is advised that while anti-TNF levels are detectable, the vaccine should be postponed^[23]^ due to the risk of developing disseminated tuberculosis.^[24]^ It is important to mention that as vedolizumab has a high intestinal specificity, vaccinations can theoretically be safer than anti-TNF agents, which provide greater systemic immunosuppression; however, it is worth noting that the effectiveness of oral vaccines is likely to be reduced.^[7,25]^

A study that evaluated the serum levels of infliximab in newborns of 11 exposed mothers found them ranging between 2.9 and 39.5 μg/mL.^[26]^ All levels were above maternal levels at delivery, and complete clearance took up to 7 months.^[26]^ Infliximab levels in newborns are associated with maternal concentration, which is dependent on the time and amount of medication administered. Maternal infliximab concentrations increased by 4.2 μg/mL at each trimester of pregnancy,^[27]^ so it is recommended that the last dose of immunobiological be given between 24 and 26 weeks of pregnancy,^[2]^ with the objective of decreasing placental transfer of medication.

In the postpartum period, some precautions must be taken, such as encouragement of breastfeeding, prophylaxis of venous thromboembolism, encouragement of adherence to medication, adequate IBD control, and mental health monitoring.^[7]^ Breastfeeding should be encouraged in all patients, given its known beneficial effects for the mother and child. Most IBD medications are considered safe, except methotrexate, tofacitinib, cyclosporine, and allopurinol. Biologics can be restarted at 24 hours after vaginal delivery or 48 hours after cesarean delivery, in the absence of infection.^[7]^ Low levels of these drugs can be found in breast milk, but without clinical repercussions.^[11]^

## 4 Conclusion

Pregnancy-related decisions, including optimal timing, should be part of the physician's discussions with women of childbearing age with IBD. Disease remission is related to favorable evolution of pregnancy and childbirth. Medications used to treat IBD are generally safe and should be maintained in most cases. There is placental transfer to the fetus in mothers exposed to infliximab, the majority of which occurs in the third trimester. Newborns of mothers exposed to infliximab may experience complications in the first year of life if exposed to live vaccines. Monitoring serum levels in the infant is useful in clinical practice and should be considered a best practice because it allows safe application of live vaccines when serum levels become undetectable, especially in countries with a high risk of infections preventable by vaccination of babies.

## Author contributions

All authors contributed to this manuscript. Maria Cecília de Aragão, Rodrigo Fedatto Beraldo, Mariana Barros Marcondes, Jaqueline Ribeiro de Barros, Giédre Soares Prates Herrerias, Rogerio Saad-Hossne, Júlio Pinheiro Baima, Ligia Yukie Sassaki contributed to the conception and design of the study; the acquisition, analysis and interpretation of data; drafting the article, revising it critically for important intellectual content, and approving the final version to be submitted.

**Conceptualization:** Maria Cecília Aragão, Rodrigo Fedatto Beraldo, Ligia Yukie Sassaki.

**Data curation:** Maria Cecília Aragão, Rodrigo Fedatto Beraldo, Mariana Barros Marcondes, Jaqueline Ribeiro Barros, Giédre Soares Prates Herrerias.

**Investigation:** Jaqueline Ribeiro Barros.

**Methodology:** Maria Cecília Aragão, Mariana Barros Marcondes, Jaqueline Ribeiro Barros.

**Project administration:** Ligia Yukie Sassaki.

**Supervision:** Rogerio Saad-Hossne, Júlio Pinheiro Baima, Ligia Yukie Sassaki.

**Validation:** Rodrigo Fedatto Beraldo, Mariana Barros Marcondes, Jaqueline Ribeiro Barros, Giédre Soares Prates Herrerias, Rogerio Saad-Hossne, Júlio Pinheiro Baima, Ligia Yukie Sassaki.

**Visualization:** Maria Cecília Aragão, Rodrigo Fedatto Beraldo, Mariana Barros Marcondes, Jaqueline Ribeiro Barros, Giédre Soares Prates Herrerias, Rogerio Saad-Hossne, Júlio Pinheiro Baima, Ligia Yukie Sassaki.

**Writing – original draft:** Maria Cecília Aragão, Rodrigo Fedatto Beraldo, Mariana Barros Marcondes, Jaqueline Ribeiro Barros, Giédre Soares Prates Herrerias, Rogerio Saad-Hossne, Júlio Pinheiro Baima, Ligia Yukie Sassaki.

**Writing – review & editing:** Maria Cecília Aragão, Rodrigo Fedatto Beraldo, Rogerio Saad-Hossne, Júlio Pinheiro Baima, Ligia Yukie Sassaki.
